# Comparison of biomechanical behavior between flat head screw and tapered head screw in internal-hex implant-abutment connection

**DOI:** 10.1038/s41598-025-23575-x

**Published:** 2025-11-13

**Authors:** Xiao Zhang, Jinyang Zhang, Yuyan Pan, Zhifa Tang, Jianyu Chen, Xianshuai Chen

**Affiliations:** 1 Guangdong Janus Biotechnology Co. Ltd, Guangzhou, 510000 China; 2 Guangdong CAS Angels Biotechnology Co. Ltd, Foshan, 528000 China; 3 Department of Oral Implantology, Guanghua School of Stomatology, Hospital of Stomatology, Sun Yat-sen University, Guangzhou, 510000 China

**Keywords:** Screw head, Biomechanical behavior, Removal torque, Friction and wear, FEA, Mechanical engineering, Implants

## Abstract

This investigation aimed to evaluate the screw head configuration on its biomechanical performance. Forty-eight screws specimens with four different screw head conical surface opening angle (β) were divided in four groups (*n* = 12): flat screw head (FHS) with β = 0°, and tapered screw head (THS) with β = 20°, 30°, and 40°. A preload torque of 35 N·cm was applied to fasten the implant-abutment assembly. The initial removal torque values (RTVs) of half the samples in each group were measured. The remaining specimens were then subjected to fatigue tests and the postload RTVs were measured. Furthermore, four 3D prosthesis models were established for FE analysis. The results suggested that the initial and postload RTVs of FHS groups were significantly lower than THS groups (*P* < .05). After mechanical cycling, the original regular raised texture features on screw head surface in FHS group were almost removed and transferred, while linear grooves appeared near the bottom of the screw head cone in THS group. The FEA results indicated that the highest stress magnitude and maximum torsion angle of the screws occurred in the FHS group. In contrast, the flat-head screw exhibited larger torque loss, greater stress concentration and more severe friction and wear.

## Introduction

Dental implant has become a widely adopted therapy for edentulous patients and are gaining increasing reliability^1^. Two stage implants are commonly used in dental implant surgery due to their high clinical success rate, which consists of a root-shaped implant surgically placed into jawbone and an abutment for retaining the upper prosthesis^2,3^. For most implant systems, the abutment and implant are mechanically integrated as a whole using an abutment screw. Therefore, the abutment screws play a very important role in the mechanical stability of the implant-abutment assembly.

Screw loosening is one of the most frequent complications occurred in clinical implant restoration. Long-term screw loosening without timely treatment may progress to more serious mechanical complications including fractures in screws, abutments, or even implants themselves^4,5^. The mechanism of screw loosening is closely related to the preload of the screw^6,7^. When the preload is applied, the screw deforms and then generate a clamping force between the implant and the abutment, thereby holding the two components together^8,9^. Once the preload gradually decreases until it is less than the separating force at the screw interface, the screw will loosen^10,11^. The main factors affecting screw preload include initial tightening torque (manufacturer’s recommended pretightening torque value), screw design (such as diameter and thread profile), screw material and surface treatment^12–16^. In order to obtain accurate target torque value in implant system, various types of screwdrivers with digital torque measuring devices were continuously introduced^17–19^. Abutment screws are usually made of titanium alloy with higher load-bearing. Obtaining a refined titanium surface with low roughness through precision machining technology is commonly employed to enhancing screw preload retention. In addition, some researchers have suggested adding surface coating material to further reduce friction and increase preload^20^.

In practical applications, the improvement of the mechanical properties of the screw is mainly considered from the above aspects. The screw head shape (conical or flat head) may also affect the reliability and the stability of the implant-abutment joint. Flat head screw is designed easier to achieve passive positioning, reducing the friction at the screw-abutment contact area, and increasing the preload at the screw thread. Some reported that conical-head screws presented higher loosening torque values compares to conventional flat-head screws^21^. Bulaqi et al. included that a higher coefficient of friction between contact surfaces in a cone screw head design leads to a lower removal torque^22^. It has also been suggested that flat head screws distribute the force more evenly within the threads and screw heads and identify non-passive casting more easily^23^. However, whether and how the shape of screw head affects the mechanical stability of implant-abutment connection remains controversial. Current research remains deficient on the impact of screw head types on the preload loss and screw loosening.

Therefore, the present investigation aims to assess the influence of screw head shape on removal torque, surface friction morphology, stress distribution and rotational angle before and after mechanical loading.

## Materials and methods

### Mechanical test

**Fig. 1:**
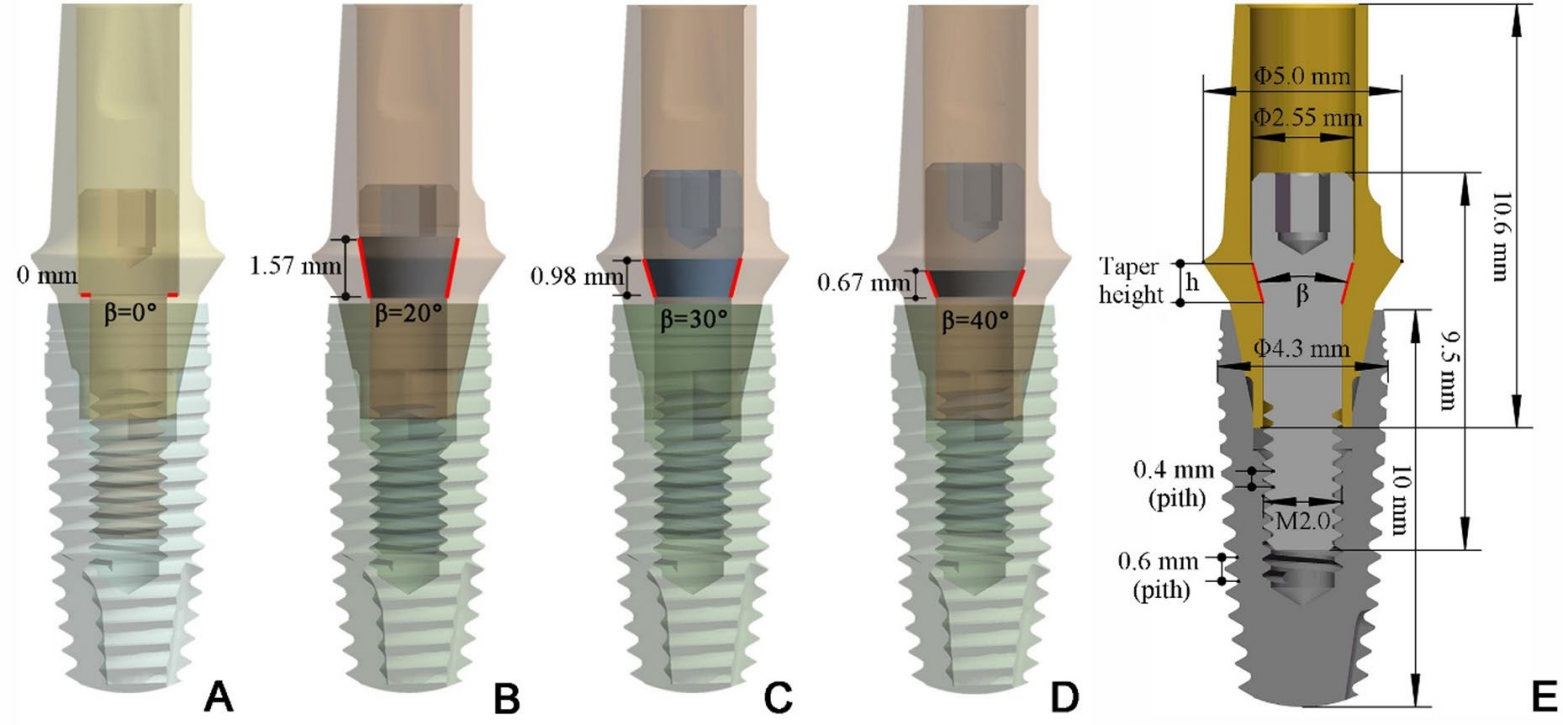
Implant-abutment assembly models with different screw head cone angle β in the present mechanical test. (**A**) β = 0° group. (**B**) β = 20° group. (**C**) β = 30° group. (**D**) β = 40° group. (**E**) Cross-sectional view of the implant-abutment assembly marked with geometric dimensions.

**Fig. 2:**
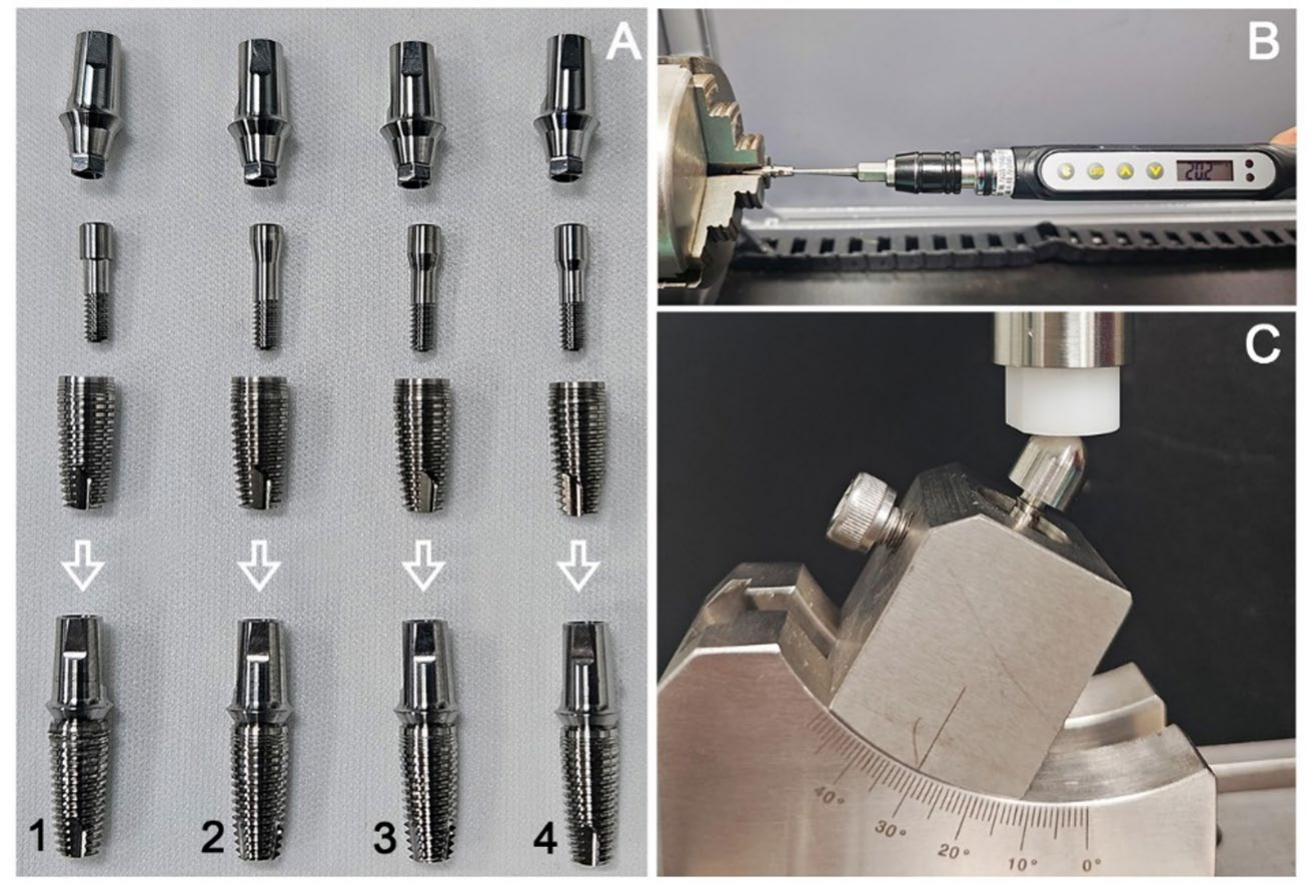
Test specimen and experimental device. (**A**) Component assembling of internal hexagon implant systems in different abutment-screw joint configuration groups: (1) FHS (flat head screw with β = 0°); (2, 3 and 4) THS (tapered head screw with β = 20°, 30° and 40°, respectively). (**B**) Digital torque tester. (**C**) Mechanical testing set-up, where the load was applied at 30° to the long axis of the implant.

Forty-eight internal hexagon implant-abutment assemblies (Angels dental implant; Guangdong CAS Angels Biotechnology Co. Ltd.) were distributed in four groups (*n* = 12/group) varying the cone angle β of abutment screw head (Fig. 1): (1) FHS (flat screw head with β = 0°); (2) THS (tapered screw head with β = 20°, 30° and 40°, respectively). All the components were processed by CNC machining center (408MT, Willemin-Macodel SA, Switzerland). The standard tolerance grade for these machined screws is IT 7. The taper tolerance of the screw head is controlled within ± 0.2°, and the accuracy grade of the screw thread is 4 h. Each set of abutment (Ti-6Al-4 V ELI, Φ5.0 mm) samples were attached to the identical bone level implants (commercially pure grade IV, Φ4.3 mm × 10 mm) by using a compatible abutment screw (Ti-6Al-4 V ELI, M2.0 × 0.4–4 h) (Fig. 2A). A manufacturer’s recommended tightening torque of 35 N.cm was applied to the screws to obtain the overall mechanical stability by a digital torque meter (Torque Meter SDE-05BN, Wiztank) (Fig. 2B). All implants were embedded into the cylindrical specimen holder (Φ15 mm×18 mm) under identical conditions using acrylic resin (XBH-30; Xinbiao Testing Instrument Manufacturing Co Ltd) with an elastic modulus close to bone. The screws were re-tightened to the same torque after 10 min to achieve the perfect preload^24^. After 5 min, half of the samples in each group were randomly selected to measure the initial RTVs. For the remaining samples, custom rigid metal caps were installed on abutments, and then the entire assemblies were mounted in Instron ES3000 (Instron, USA) for dynamic mechanical cycling under a load of 250 N with a frequency of 15 Hz (Fig. 2C). After a given 1 million mechanical cycles, the postload RTVs were measured and recorded. To evaluate and compare the wear pattern of the screw head cone area, the friction and wear morphology of screw head fitting surface before and after mechanical cycling were analyzed using confocal laser microscope (Keyence, VK-X1050 Series).

### Statistical analysis

Statistical analysis was conducted by using Origin data processing software (v9.0, OriginLab Corporation), with significance level was set at *P<*.05. The Shapiro–Wilk test was used to verify the normality of the recorded RTVs of all screws for pre- and post-mechanical fatigue testing. One-way ANOVAs were performed on initial and postload RTVs data of each group, the multiple comparisons of means were analyzed with Tukey test.

### Finite element analysis

**Fig. 3:**
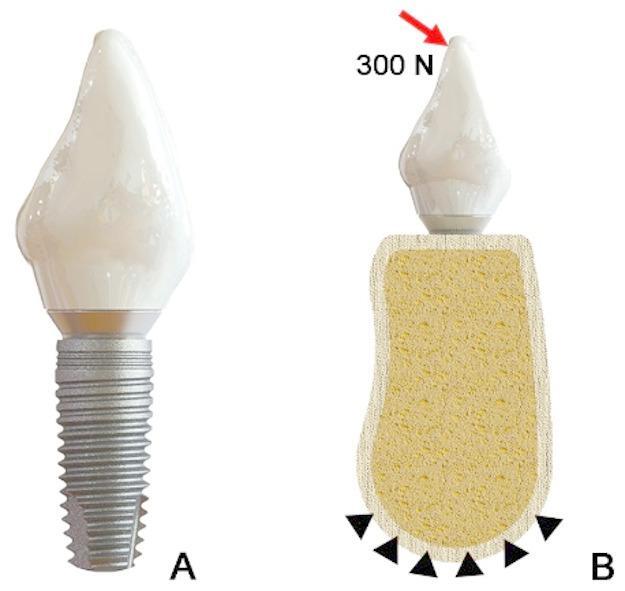
Finite element models. (**A**) Implant-abutment complex model with a cement-retained crown over abutment. (**B**) Complete restoration model with occlusal load (300 N) at the crown apex and full constraint on the alveolar bone surface.

Four virtual implant-abutment complex models were built using 3D modeling software SolidWorks (SolidWorks v12.0; Dassault Systèmes). The implant and abutment were assembled together by applying a preload torque of 35 N.cm to the abutment screw. All the models were imported into the FEA software (ANSYS, ANSYS Inc.) and meshed with 10-node second-order tetrahedral element. The mesh size of implant system components is finally optimized to 0.3 mm to achieve the convergence condition of the system stress change. The screw-abutment and screw-implant mating interface were set as friction contact with a friction coefficient of 0.2.^22,25^. In this analysis, all components are assumed to be homogeneous, continuous, isotropic, and linear elastic materials. The material properties of the models were established based on exiting literature (Table 1)^25^. A static load of 300 N was applied lingually at the crown apex at 30° to the long axis of the implants to simulate the actual bite force (Fig. 3)^26^. The stress distribution of abutment screws in FHS and CHS groups were calculated to compare the stress difference. In the case of a given preload torque, the screw needs to be rotated at a certain angle to achieve a specific tightening effect. In order to anticipate the potential clamping capacity between screw head and abutment contact surface, the overall rotation angles (θ) relative to the screw’s long axis were measured at a selected conical surface of the screw head and calculated using ANSYS. The output result type was the “relative rotation” during loading in the pre-tightening direction.

**Table 1:** Materials properties of the finite element models.

Part	Materials	Young’s modulus (GPa)	Poisson’s ratio
Implant	Titanium grade 4	105	0.34
Abutment, screw	Ti-6A1-4 V	110	0.32
Alveolar bone	Cortical bone	14	0.3
	Cancellous bone	1.4	0.3
Crown	Zirconia	205	0.19

## Results

**Fig. 4:**
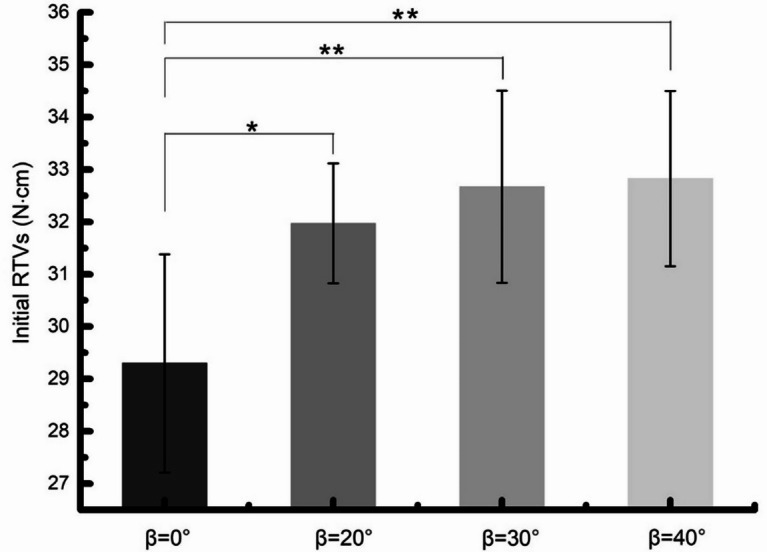
Means and 95% confidence intervals of initial RTVs of all groups. FHS group and THS group statistically significantly different, β = 0° vs. β = 20°: *P* = .0362, β = 0° vs. β = 30°: *P* = .0062, β = 0° vs. β = 40°: *P* = .0041.

**Fig. 5:**
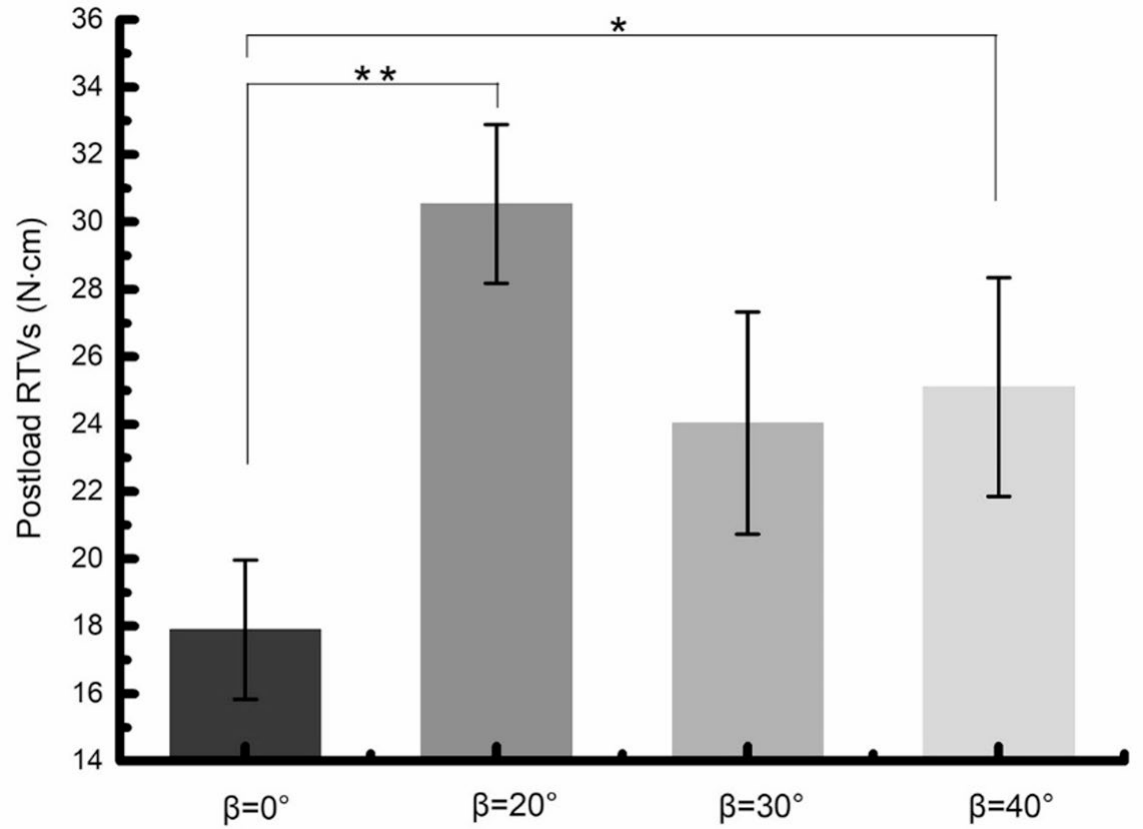
Means and 95% confidence intervals of postload RTVs of all groups. FHS group and THS group statistically significantly different, β = 0° vs. β = 20°: *P* = .0024, β = 0° vs. β = 40°: *P* = .0433.

The initial and postload RTVs of the 4 groups were recorded and analyzed statistically, with a range of 29.30 ± 2.08 to 32.82 ± 1.67 N.cm and 17.9 ± 1.19 to 30.53 ± 1.36 N.cm, respectively (Table 2). There were significant differences in initial and postload RTVs between FHS group and THS group before and after mechanical cycling (*P* = .0025, *P* = .0040). The initial and postload RTVs of FHS group (β = 0°) were significantly lower than THS groups (β = 20°, 30° and 40°). However, for THS groups, no significant differences were found among the 20°, 30°, 40° groups (Figs. 4 and 5).

**Table 2:** RTVs in all groups before and after mechanical cycling.

	Group	Number of screws	Mean ± SD RTVs (*N*.cm)	F	*P*
Before mechanical cycling	0°	6	29.30 ± 2.08	6.356	0.0025
	20°	6	31.97 ± 1.14		
	30°	6	32.67 ± 1.83		
	40°	6	32.82 ± 1.67		
After mechanical cycling	0°	6	17.9 ± 1.19	10.311	0.0040
	20°	6	30.53 ± 1.36		
	30°	6	24.03 ± 1.90		
	40°	6	25.10 ± 1.87		

Values are recorded as mean ± standard deviation, α = 0.05, one-way ANOVA.

**Fig. 6:**
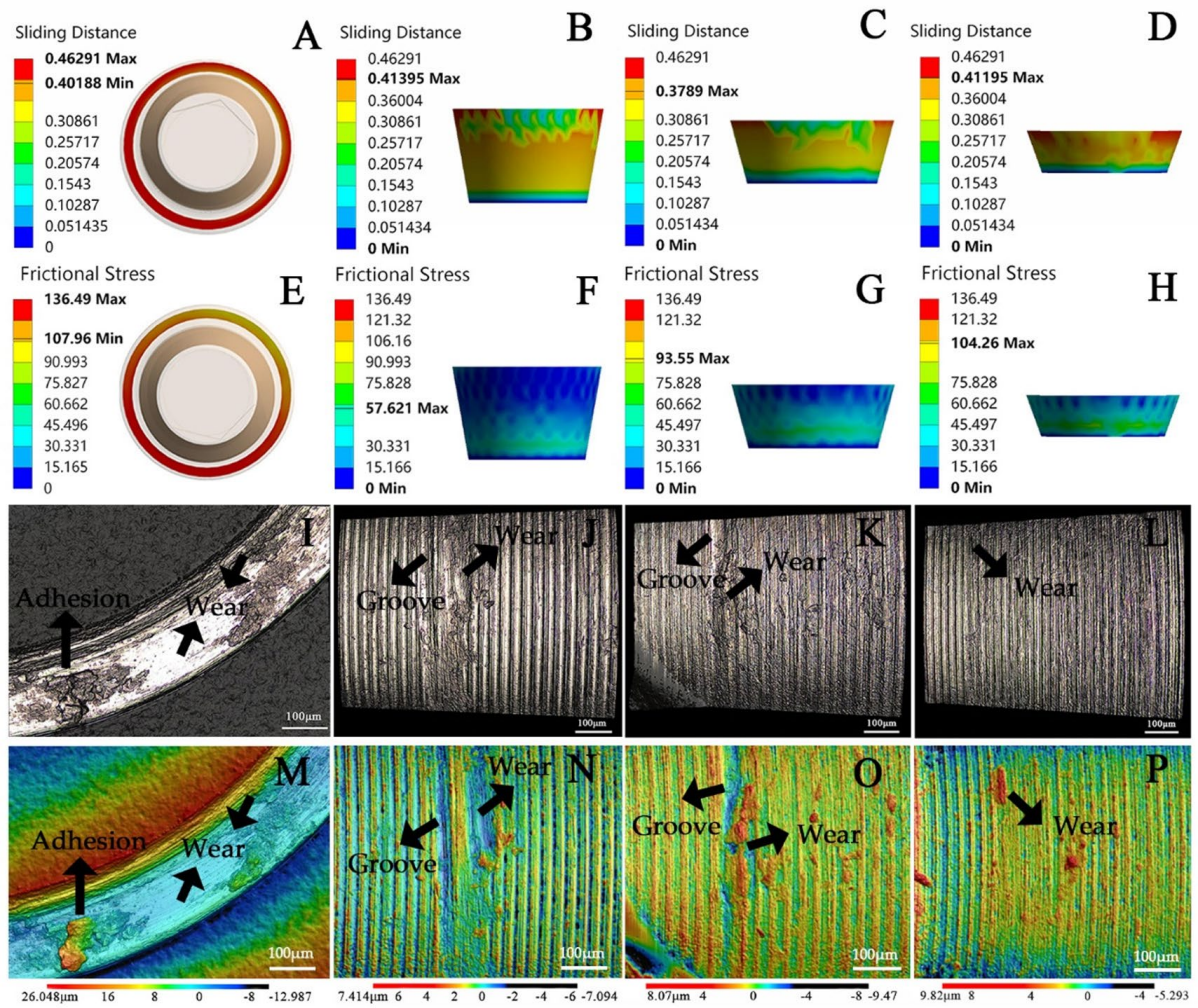
The sliding distance cloud map, frictional stress nephogram and wear-induced micrograph of the screw head contact surface after mechanical cycling. (**A**–**D**) Sliding distance in β = 0°, β = 20°, β = 30°, β = 40° groups, respectively. (**E**–**H**) Friction stress in β = 0°, β = 20°, β = 30°, β = 40° groups, respectively. (**I**) Stereo micrograph of β = 0° group indicates that the regular raised texture features on the metal surface left by machining were almost completely removed and transferred, resulting in a large areas of accumulation and adhesion containing metal debris remained on the circular contact surface. (**J**–**L**) Stereo micrograph of β = 20°, β = 30° and β = 40° groups, showing that the wear patterns were mainly manifested as linear grooves near the bottom of the cone. (**M**–**P**) Magnified 3D color micrograph of β = 0°, β = 20°, β = 30° and β = 40° groups, describing the difference in height in color.

**Fig. 7:**
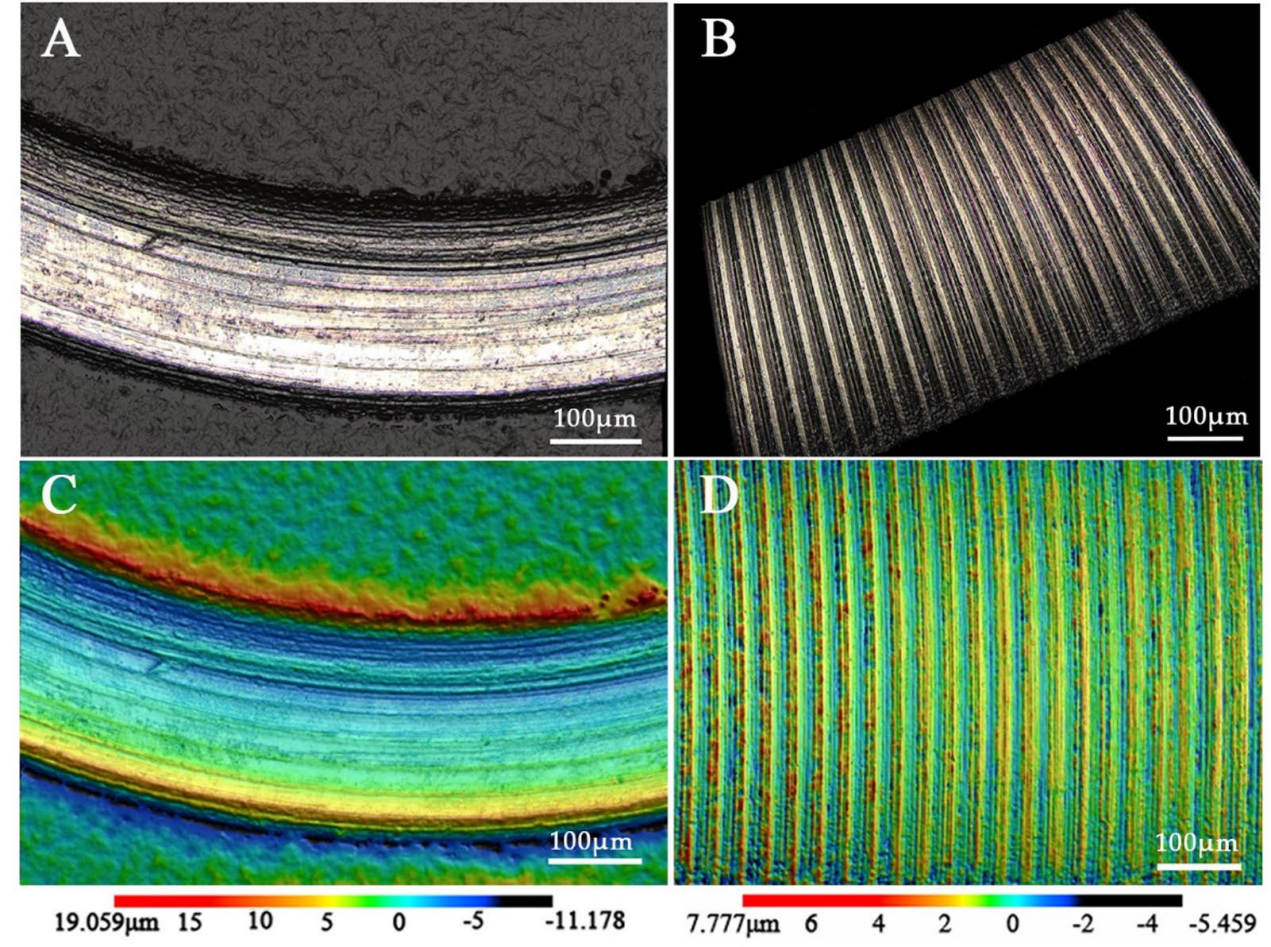
Micrograph of screw head contact surface before mechanical cycling. (**A** and** B**) Microscope image of β = 0° group and β = 30° group, illustrating the regular and even stripes and concavity features left on the surface by machining. (**C** and **D**) Enlarged 3D color surface topography of β = 0° group and β = 30° group, describing the height difference in height with color.

After mechanical cycling, different degrees of friction and wear occurred on the contact surface of the screw head in FHS group and CHS group (Fig. 6). For the FHS group, the original regular and uniform stripes and concavity texture on the circular contact surface of the screw head were almost removed, with a large area of accumulation and adhesion of metal debris was generated (Fig. 6I). Linear grooves near the bottom of the screw head cone and local wear in the were found CHS group (Fig. 6J, K and L). The microscopic findings were comparable to the sliding distance (Fig. 6A, B, C and D) and frictional stress peak concentration in the virtual screw head cone (Fig. 6E, F, G and H). The entire contact surface of the FHS group screw head almost completely experienced sliding friction and exhibited the maximum sliding distance of 0.46 mm. In addition, the enlarged 3D color micrograph describes the height difference with color, which further visually display the detailed characteristics of surface wear (Fig. 6M, N, O and P). Figure 7 shows the microscope image of screw head before mechanical cycling, revealing regular and even stripes and concavity features left on the surface after machining, with little surface friction and wear observed.

**Fig. 8:**
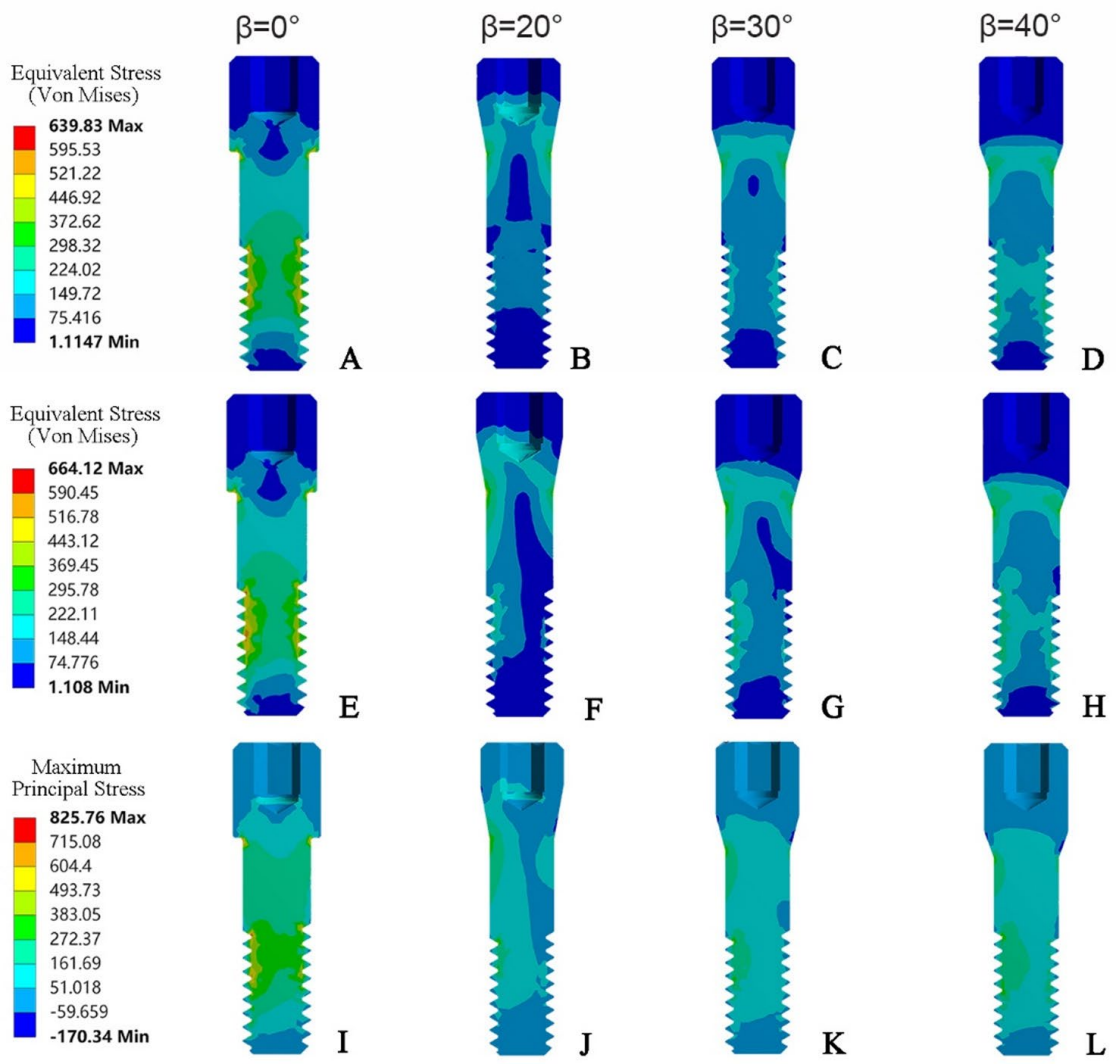
The stress distribution of the screws. (**A**–**D**) von Mises stress after application of preload torque in β = 0°, β = 20°, β = 30°, β = 40° groups. (**E**–**H**) von Mises stress after application of occlusal load in β = 0°, β = 20°, β = 30°, β = 40° groups. (**I**–**L**) principal stress after application of occlusal load in β = 0°, β = 20°, β = 30°, β = 40° groups.

**Fig. 9:**
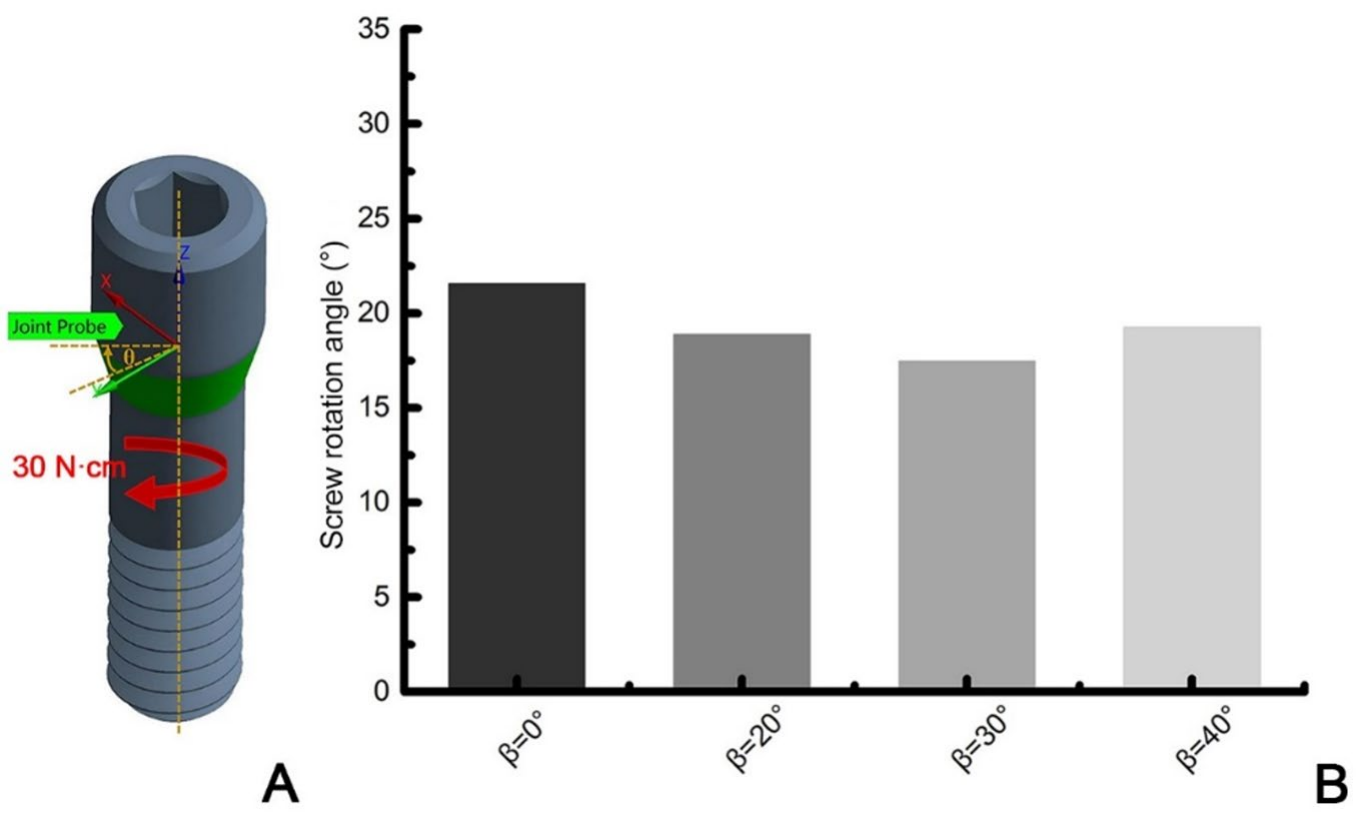
(**A**) Schematic diagram of rotation angles (θ) of screw head relative to its long axis. (**B**) The calculated rotation angles values.

The stress distribution in the screws with identical preload torque before and after the application of an occlusal load were shown in Fig. 8. It can be observed that the stress distribution extends from the screw head to the engaged thread region. Before the application of external loading, the stress distribution was symmetrical. After the occlusion load was applied, the stress distribution then became asymmetrical. Stress concentration predominantly occurred on the tensile side and the stress on the tensile side was higher than that on the compressive side. The maximum principal stress (PS) was compared with the maximum equivalent Von-Mises stress (VMS) to better understand the stress distribution of the screws. The maximum values of VMS and PS were observed in both the FHS group, which were 664.12 MPa and 825.76 MPa respectively. The VMS and PS ranges of the THS group screws were 394.25 to 428.41 MPa and 421.12 to 439.06 MPa, respectively. Additionally, a high stress concentration was also found in the first engaged thread region of FHS group. The rotation angles θ of the abutment screws were quantified within 17.45–21.55 degrees (Fig. 9), with maximum rotation angle value (21.55 degree) occurring in the FHS group.

## Discussion

The results indicated that there were significant differences in initial and postload RTVs between FHS group and THS group. Compared with THS group, the removal torque values of FHS group decreased by 8.35% to 10.7% and 25.5% to 41.3% before and after 1 million mechanical cycles. It is noteworthy that no significant difference was observed in initial and postload RTVs among the β = 20°, 30°, 40° groups. Coppedê et al.^21^ conducted a comparative analysis of the loosening torque for flat-head abutment screw and conical screw, specifically focusing on with external-hex and inter tri-channel platform connections. The authors concluded that conical-head screws exhibited higher loosening torque values compared to flat-head screws pre- and post-loading. Nevertheless, their studies only selected conical head screws with a single conical angle of 50°, which has certain limitations. The latest research by Szajek et al.^23^ demonstrated that an abutment-fixture butt joint and a tapered screw were identified as key geometry features that minimize the drop in screw preload. Regardless of the implant-abutment interface configuration, consistent result can be obtained that flat head screws are more likely to lose preload than conical head screws.

Titanium screw exhibits strong adhesion in sliding contacts against titanium abutment, resulting in high friction and severe wear^27^. Severe wear between the abutment and screw contact surface induced by adhesion after mechanical loading may lead to early failure, including preload reduction, screw loosening, contact-induced surface cracks and screws fracture^28^. Therefore, the friction and wear morphology of the screw head should be given sufficient attention.

Laser microscope image revealed that friction and wear occurred on the contact surface (abutment-screw mating surface) of the screw head in all groups after mechanical cycling. Large areas of accumulation and adhesion containing metal debris were observed in the FHS group, presenting significant contrast to the surface texture morphology without mechanical loading. During cyclic loading, large micro-motion and friction occur on the abutment-screw matching surface, resulting in plastic deformation of the regular raised texture features of the metal surface left by machining, which were almost completely removed and transferred. For the THS group, the screw head wear was mainly manifested as linear grooves (about 20 μm in width) near the bottom of the cone (where the abutment-screw contact area is about to end) and localized wear. This may be due to the surface fatigue wear of the conical surface under the repeated cyclic contact compressive stress.

In comparison, the contact surface of flat-head screws in FHS group exhibits the most severe friction wear. This is consistent with the FEA findings that the contact surface friction stress and sliding distance of the screw head in FHS group were both the highest (136.49 MPa and 0.46 mm), which were 23.6% ~ 57.7% and 10.9% ~ 19.6% higher than THS group, respectively. In addition, the friction stress concentration was basically consistent with the wear area observed in the microscopic images. From a mechanical design perspective, the abutment-screw taper joint could form a friction-locking effect between conical mating surfaces, thereby resisting the rotational micro-movement of the screw during mechanical loading, reducing torque loss and friction wear^29^.

It has been proved that rotational micromovements and slipping on the contacting surfaces are the primary reasons associated with screw preload loss^30^. The ideal connection between the screw and the abutment after applying a preload is intended to be fixed and motionless (only static friction is experienced).Unnecessary relative rotation could result in sliding contact, inevitable dynamic friction and wear, thereby inducing loosening or even early failure of the screw-abutment joint^31,32^. The magnitude of the output rotational angle along the pre-tightening direction can be used as an indirect indicator of clamping force^13^. The simulation results of screw rotation angle showed that the calculated rotation angle of FHS group (21.5 degrees) was significantly higher than THS group (17.45 to 19.25 degrees). Similar measurements of rotation angles (14.9 ± 1.5 ° to 17.0 ± 3.1 °) for commercial titanium alloy screws at 32 N cm torque have been reported by Martin et al.^13^ It can be inferred that the tapered-head screw is less prone to rotational micro-motion than the flat-head screws, thus generating a greater clamping force make the screw stable against external forces.

An abutment-screw mechanical joint with a flat-head screw will result in high stress distribution and friction stress level at the screw head. When occlusal loads are applied, the tapered head screw tends to produce a large frictional resistance to resist the rotational micromovements of the screw in the direction of loosening, thereby maintaining the clamping force and preload and preventing the screw from loosening. In addition, the other two aspects affecting screw preload loss should also be further considered. On the one hand, excessive loading may cause elastic rebound of the accumulated torque in the screw shank and result in preload loss^33^. On the other hand, pure titanium implants are prone to gradual deformation during actual loading due to their relatively low strength grade. In this case, a high stress can be generated at the neck of flat-head screw, which may even exceed its yield strength. This may further lead to local geometric changes and screw shank shortening, and facilitate screw loosening.

The present studies have some limitations. Firstly, when a screw is secured with a certain pre-tightening torque to lock the implant-abutment connection, contact forces and frictional micromovements occur primarily at two interfaces: between the screw head and abutment, and between the screw threads and the implant internal threads^17^. although the configuration of the screw thread in all groups was identical. The wear patterns and roughness changes of flat head and conical head screw threads after mechanical cycling need further study to prove the reliability of the conclusions. Secondly, finite element analysis requires more refined model processing and algorithm optimization to obtain more accurate results, such as model detail processing and model accuracy verification. Thirdly, the deviation of the screw axis before and after mechanical testing may have a certain impact on the experimental results. The deviation of the screw axis after mechanical testing should be further confirmed. Finally, the small sample size may lead to deficiencies such as low test power in statistical analysis. It is necessary to further expand the sample size to improve the reliability of the conclusions.

## Conclusion

Compared to the tapered head screw, the flat-head screw exhibited greater initial and postload RTVs, higher stress concentration levels and larger rotation angles. The tapered-head screw tends to produce a large friction resistance to resist the rotational micro-movements, thereby reducing preload loss and maintaining mechanical connection stability.

## Data Availability

The datasets generated during and/or analysed during the current study are available from the correspondingauthor on reasonable request.
